# Cesarean section in a high-parity community in Saudi Arabia: clinical indications and obstetric outcomes

**DOI:** 10.1186/1471-2393-14-92

**Published:** 2014-02-28

**Authors:** Mohammed A Al Rowaily, Fahad A Alsalem, Mostafa A Abolfotouh

**Affiliations:** 1 Department of Family Medicine and Primary Health Care, King Abdulaziz Medical City, National Guard Health Affairs, Riyadh, Saudi Arabia; 2 Department of Obstetrics and Gynecology, King Abdulaziz Medical City, National Guard Health Affairs, Riyadh, Saudi Arabia; 3 Biobanking Section, King Abdullah International Medical Research Center (KAIMRC), Riyadh, Saudi Arabia; 4 King Saud Bin-Abdulaziz University for Health Sciences (KSAU-HS), National Guard Health Affairs, POB 22490, Riyadh 11426, Saudi Arabia

**Keywords:** Cesarean section, Indications, Maternal/fetal, Outcomes, Near miss, Saudi Arabia

## Abstract

**Background:**

The study of the indications for cesarean section (CS) and its outcomes are useful for hospitals, clinicians, and researchers in determining strategies to lower the primary and repeat CS rate. The aim of this study was to identify the indications for CS and the incidence of adverse maternal/fetal outcomes in a tertiary care setting.

**Methods:**

A retrospective cohort study of women (n = 4305) who gave birth by CS at King Abdulaziz Medical City (KAMC), Riyadh, Saudi Arabia (June 2008 to February 2011), was performed. All of the women’s medical records were reviewed by two consulting physicians to obtain the primary indications for CS and determine the maternal characteristics, type of CS (emergency or elective), and birth weight. All adverse maternal and fetal outcomes were recorded. The point and interval estimates of the odds ratios were calculated using a logistic regression model to identify the significant predictors of adverse maternal and/or fetal outcomes.

**Results:**

Of a total of 22,595 deliveries from 2008 to 2011, 4,305 deliveries were CS deliveries (19.05%). Two-thirds (67%) of all CS deliveries were emergency CSs, and the remaining deliveries were elective CSs (33%). Difficult labor (35.9%), fetal distress (21.9%) and breech presentation (11.6%) were the most frequent indications of emergency CS, while previous CS (54.3%), breech presentation (20.4%) and maternal request (10.1%) ranked first for elective CS. Adverse maternal and fetal outcomes were diagnosed in 5.09% and 5.06% of deliveries, respectively, with a significantly higher incidence in the emergency (6.06% & 5.51% respectively) than in elective CS (3.10 & 4.16% respectively). Blood transfusion was the most frequent adverse maternal outcome (3.72%), followed by ICU admission (0.63%), HELLP (0.51%), and hysterectomy (0.30%), while IUGR (3.25%) was the most frequent adverse fetal outcome, followed by IUFD and the need for ICU admission (0.58% each). Adverse maternal outcomes were significantly predicted by high gravidity (OR = 2.84, 95% CI:1.26-6.39, p = 0.011) and preeclampsia (OR = 2.84, 95%CI:1.83-4.39, p < 0.001), while adverse fetal outcomes were predicted by: twinning (OR = 1.81, p = 0.002), hydramnios (OR = 6.70, p < 0.001), and preeclampsia (OR = 2.74, p < 0.001). Preterm delivery was a significant predictor for both adverse maternal and fetal outcomes (OR = 2.39, p < 0.001 & OR = 4.57, p < 0.001, respectively).

**Conclusions:**

Difficult labor and previous CS were the main indications for CS in Saudi Arabia. High gravidity was a significant predictor of adverse maternal outcomes. Encouraging Saudi women to consider embarking on fewer pregnancies could act as a safeguard against mandatory CSs for subsequent births in multigravida and grand-multigravida Saudi females. Future prospective study that addresses women with repeat CSs and their association with adverse maternal and fetal outcomes is recommended.

## Background

During the past several decades, cesarean section (CS) has become a common operative procedure, with the proportion of women giving birth by CS increasing over time in all developed countries. In 2007, 30.9% of Australian women gave birth by CS, which was increased from 21% in 1998 [1]. In the USA, a similar increase was reported, where 31.1% of all births were delivered by CS in 2006, which was increased from 20.7% in 1996 [2]. In the UK, the overall rate of cesarean birth is lower, accounting for nearly 25% of all births from 2007 to 2008; however, this rate has increased by approximately 50% from 1995–1996 [3]. In Europe, the rates vary considerably, with rates of 15% in Norway and The Netherlands, approximately 17% in Sweden and Finland, and 37.8% in Italy [4]. In Saudi Arabia, the CS rate (CSR) accounts for approximately 10% of all births, reaching 20% in tertiary centers [5].

The observed increase in cesarean birth has been attributed to a number of factors, including advanced maternal age, particularly with the first birth, multiple pregnancies, breech presentation, suspected low infant birth weight, private hospital status [6], and an increasing maternal BMI [7]. Other factors include organizational factors, the woman's choice regarding childbirth and preferences for care, and the obstetrician's characteristics and care practices [8].

CS constitutes a major surgical procedure and, as such, is associated with a number of surgical complications. Despite this, a proportion of women are electing to have their babies delivered in this manner without any other indications [9]. It has been clearly proven that an emergency CS is associated with a substantial morbidity and mortality rates [10]. In the UK during 1994-1996, maternal mortality was found to be 5.9 per 100,000 completed pregnancies for elective CS delivery versus 18.2 for emergency CS; maternal mortality for vaginal birth was 2.1 per 100,000 completed pregnancies [11]. By having an elective CS, any woman can make sure that she does not run the medical and psychological risks of an emergency CS, which have become more prominent recently [11]. However, it has been reported that an elective CS is less safer than a vaginal delivery, with regard to maternal mortality [12,13].

High parity (5+) is common in developing countries, particularly in Arab nations such Saudi Arabia, where large families are the norm [14]. The association between multiparity and pregnancy outcomes has been studied extensively [15-17]. Repeat CS is a common phenomenon in the KSA, most likely due to cultural opposition to CS limitation, as this would limit the size of the family [18]. The CS will continue to increase due to the strong wish to have large families in Saudi Arabia and the self-perpetuating nature of CS. The aims of this study were: 1) to identify the clinical indications for CS deliveries in a tertiary hospital serving a high-parity community, 2) to determine incidence of adverse maternal and fetal outcomes in elective and emergency CS deliveries, and 3) to determine the significant predictors of these outcomes.

## Methods

### Design

This study was a retrospective cohort study consisting of 4,305 women who gave birth by CS.

### Setting

King Abdulaziz Medical City (KAMC), Riyadh Saudi Arabia, is a tertiary care hospital that serves a steady population of the national guard, military personnel, and civilians and their dependents. All deliveries are hospital based and attended by midwives, and the information was documented by the attending staff. The decision to undertake CS was made by a specialist in every case. The procedure itself was performed by specialists or by residents under the specialist’s supervision. The hospital stay duration was a minimum of 3 days. Therefore, the data include all CS hospital deliveries.

### Data collection

All medical records of the women who delivered by CS between June 1, 2008, and February 2011 at KAMC were reviewed by two consulting physicians to obtain the primary indication for CS, and the following information was extracted: maternal characteristics, such as maternal age, parity (including the current delivery), previous abortions, previous still births, number of live births, gestational age (measured according to the last menstrual period), birth weight, attendance for antenatal care, type of delivery (emergency or elective CS), the number of previous CSs, indication for CS, and birth weight. All adverse maternal and fetal outcomes were recorded. Adverse maternal outcomes included near miss or severe acute maternal morbidity (near miss/SAMM) and maternal death. Adverse fetal outcomes included intrauterine fetal death (IUFD), neonatal death, the need for ICU admission, IUGR, and intrauterine transfusion.

The indications were grouped hierarchically into the following 11 diagnostic categories for both elective and emergency CS [19]: 1) difficult labor or dystocia, 2) fetal distress, 3) previous CS, 4) breech presentation, including frank breech, complete breech, or incomplete breech, with either dystocia, fetal distress, none or both, or recognizing that the breech presentation was a cause of both dystocia and fetal distress, 5) delivery of a premature fetus, 6) antepartum hemorrhage (i.e., placental abruption or placenta previa), 7) delivery of twins/triplets, 8) cord prolapse, 9) maternal conditions (i.e., perineal repair, failed induction, and others), 10) fetal conditions (fetal macrosomia, transverse lie, and fetal anomalies), and 11) maternal request.

### Operational definitions

Planned operations - at a time that suited the woman and the maternity team- were considered “elective”, whereas all other operations were considered “emergency” [20]. The CSR is typically defined as the number of cesarean deliveries over the total number of live births and is usually expressed as a percentage. However, in the present study, only the proportion of CS deliveries as a percentage of total deliveries was estimated. Parity was classified as nulliparous (no previous viable pregnancy), multiparous (given birth to 1–4 children), and grand-multiparous (given birth to 5+ children) [21]. A stillborn baby was defined as a baby born after the 24th week of pregnancy who did not show any signs of life. If the baby died in the womb, it was considered an intra-uterine stillbirth. If the baby died during labor, it was considered an intra-partum stillbirth. If the baby died before 24 weeks, it was considered a miscarriage. Fetal distress was determined by an abnormal fetal heart rate pattern diagnosed either by auscultation, continuous electronic fetal monitoring (EFM), or signs of serious deficiency of the placenta, such as severe intrauterine growth retardation. Dystocia was characterized by the slow and abnormal progression of labor. Because dystocia rarely can be diagnosed with certainty, the imprecise term “failure to progress” has been used, and this term includes the lack of progressive cervical dilation, the lack of descent of the fetal head, or both [22]. HELLP syndrome is a serious complication in pregnancy characterized by hemolysis, elevated liver enzymes, and low platelet count. It is a recognized complication of preeclampsia and eclampsia (toxemia) during pregnancy [23]. Women who experienced and survived a severe health condition during pregnancy, childbirth or postpartum, such as: hysterectomy, blood transfusion, ruptured uterus, retained placenta, HELLP syndrome, pulmonary embolism, renal failure, heart failure and pelvic abscess, are considered as near miss or severe acute maternal morbidity (SAMM) cases [24,25].

### Statistical analysis

Statistical analysis was conducted using SPSS (Statistical Package for the Social Sciences) software. Descriptive measures, such as the arithmetic mean and standard deviation, were used to describe quantitative data. For quantitative data, Student’s *t*-test was used to compare the sample means. Pearson’s chi-square test was used to compare categorical data. The point and interval estimates of the odds ratios were calculated using a logistic regression model including all the variables in the analysis. The group with a lower risk of adverse maternal and/or fetal outcomes was used as the reference category for each variable. All variables of known importance were included in the model, even if they were not associated with adverse outcome in an rx2 table, to control for their potential confounding effects. All variables were categorically represented, as the effects of the quantitative variables on the outcome were unlikely to be linear or showed no linear trends. Statistical significance was set at P < 0.05.

### Ethical consideration

The study protocol was approved by the Institutional Review Board of The Saudi Ministry of National Guard, Health Affairs, Riyadh, Saudi Arabia. (Ref. #RR07/34).

## Results

Of a total of 22,595 deliveries from 2008 to 2011, 4,305 deliveries were CS deliveries, with a rate of 19.05%. Of all the CS deliveries, two-thirds (67%) were emergency CSs, and one-third were elective CSs (33%). The women ranged in age from 15 to 48 years, with a mean age of 30.86 ± 6.36 years. More than half (52.7%) of women were of gravid 4 or more, with a mean gravidity of 4.60 ± 3.33. Grand-multiparity (5+ births) was prevalent in 27.3% of all women, with an average parity of 2.98 ± 88. The mean gestational age was 37.97 ± 2.68 weeks, and the mean birth weight was 3016.88 ± 667.27 grams. Women with elective CSs showed a significantly higher mean age (32.77 ± 5.70 versus 29.88 ± 6.46 years, *t* = 15.01, p < 0.001), mean gravidity, mean parity, and mean birth weight in grams, whereas women with emergency CSs showed a significantly higher gestational age in weeks.

More than one-third of the women (37.2%) had experienced one or more abortions and/or stillbirths; 5% gave birth to twins. Additionally, 14.4% had one or more co-morbidity; 18.8% of patients had GDM, 5.7% had antepartum hemorrhage, 4.9% had preeclampsia, and 5.5% had hydramnios. Women who opted for elective CS showed significantly higher rates of previous abortions and/or still births, twinnings, and GDM, whereas women who underwent emergency CS showed higher rates of antepartum hemorrhage, preeclampsia, and hydramnios. Moreover, a significantly higher proportion of the women who underwent emergency CS did not receive complete antenatal care (Table 1).

**Table 1:** Demographic and obstetric characteristics of women who gave birth by cesarean section in central Saudi Arabia

**Characteristics**	**Emergency (n = 2886, 67.0%)**		**Elective (n = 1419, 33.0%)**		**Total (n = 4305, 100.0%)**		**Significant difference**
	No.	%	No.	%	No.	%	
*Age group (years)*							
<20	96	3.3	12	0.8	108	2.5	
20-24	559	19.4	107	7.5	666	15.5	*χ*2 = 204.82, p < 0.001**
25-29	794	27.5	290	20.4	1084	25.2	
30+	1437	49.8	1010	71.2	2447	56.8	
Mean (SD)	29.94 ± 6.47		32.73 ± 5.72		30.86 ± 6.36		*t* = 14.38, p < 0.001**
*Gravidity*							
One	744	25.8	105	7.4	849	19.7	
Two	507	17.6	144	10.1	651	15.1	*χ*2 = 302.02, p < 0.001**
T*hree*	340	11.8	198	14.0	538	12.5	
Four or more	1295	44.9	972	68.5	2267	52.7	
Mean (SD)	4.18 ± 3.34		5.47 ± 3.12		4.60 ± 3.33		*t* = 12.44,p < 0.001**
*Parity*							
Nulliparity (0 births)	907	31.4	135	9.5	1042	24.2	*χ*2 = 253.73, p < 0.001**
Multiparity (1-4 births)	1295	44.9	794	56.0	2089	48.5	
Grand multiparity(5+ births)	684	23.7	490	34.5	1174	27.3	
Mean (SD)	2.59 ± 2.89		3.75 ± 2.70		2.98 ± 2.88		*t* = 12.92, p < 0.001**
*Gestational age*							
<37 weeks (preterm)	610	21.1	172	12.1	782	18.2	
37-42 weeks (term)	2156	74.7	1236	87.1	3392	78.8	*χ*2 = 96.90, p < 0.001**
>42 weeks (postterm)	120	4.2	11	0.8	131	3.0	
Mean (SD)	38.07 ± 3.08		37.77 ± 1.56		37.97 ± 2.68		*t* = 4.17, p < 0.001**
*Previous abortion/stillbirth*	988	34.8	615	42.0	1603	37.2	*χ*2 = 21.86, p < 0.001**
*Twinning*	125	4.3	92	6.5	217	5.0	*χ*2 = 9.21,p = 0.002**
Birth weight (g)							
<2500 (LBW)	560	19.4	156	11.0	716	16.6	
2500-3500 (normal)	1638	56.8	1019	71.8	2657	61.7	*χ*2 = 94.79, p < 0.001**
>3500 (large)	688	23.8	244	17.2	932	21.6	
Mean (SD)	2997.32 ± 730.89		3056.65 ± 512.09		3016.88 ± 667.27		*t* = 3.09, p = 0.002**
*Antenatal care*	2368	83.3	1378	94.2	3746	87.0	*χ*2 = 100.97, p < 0.001**
*GDM*	503	17.4	304	21.4	807	18.8	*χ*2 = 9.90, p = 0.002**
*Maternal comorbidity*	376	13.1	244	17.0	620	14.4	*χ*2 = 11.69, p = 0.001**
*Antepartum hemorrhage*	235	6.6	11	4.0	246	5.7	*χ*2 = 11.63, p = 0.001**
*Preeclampsia*	199	6.7	19	1.4	209	4.9	*χ*2 = 58.36, p < 0.001**
*Hydramnios*	172	6.1	63	4.2	235	5.5	*χ*2 = 6.40, p = 0.011**

*χ*2: Chi-square test, *t*: Student’s t-test, **: Statistically significant difference.

### Indications for CS

**Figure 1:**
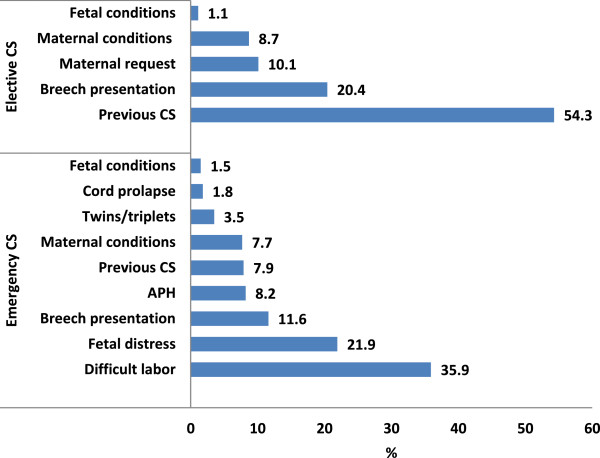
**Indications for CS at King Abdulaziz Medical City, Riyadh, Saudi Arabia.***Note: Fetal conditions include macrosomia, transverse lie and very premature fetus.*

Difficult labor and previous CSs (24% and 23.2%, respectively) ranked first as indications for CS, followed by fetal distress and breech presentation (14.7% and 14.5%, respectively), maternal conditions (8%), APH (5.5%), multiple delivery (4.1%), and maternal request (3.3%); fetal conditions and cord prolapse were indications in 1.3% and 1.2%, of the deliveries, respectively. Difficult labor (35.9%), fetal distress (21.9%) and breech presentation (11.6%) were the most frequent indications of emergency CS, while previous CS (54.3%), breech presentation (20.4%) and maternal request (10.1%) were ranked first for elective CS. (Figure 1).

### Adverse obstetric outcomes of CS

**Figure 2:**
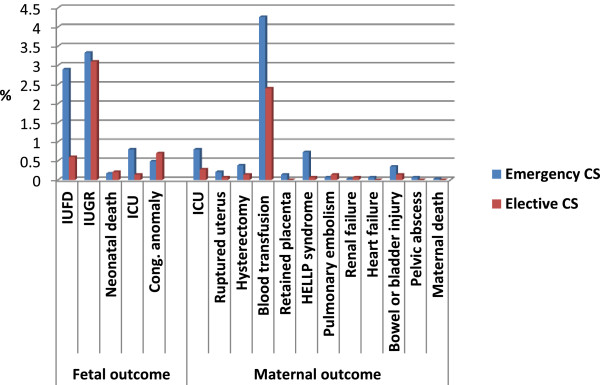
Incidence of adverse maternal and fetal outcomes in emergency and elective CS at King Abdulaziz Medical City, Riyadh, Saudi Arabia.

Adverse maternal outcomes were diagnosed in 219 women, resulting in an incidence rate of 5.09 per 100 CS women. This rate was significantly higher among women who underwent emergency CS (6.06%) than those who underwent elective CS (3.10%) (*χ*2 = 17.30, p < 0.001). Blood transfusion was the most frequent adverse maternal outcome (3.72%), followed by ICU admission (0.63%), HELLP (0.51%), and hysterectomy (0.30%). Additionally, adverse fetal outcomes were diagnosed in 218 births, with a rate of 5.06 per 100 CS women. Again, this rate was significantly higher among the emergency CSs (5.51%) than the elective CSs (4.16%), (*χ*2 = 3.61, p = 0.029). IUGR (3.25%) was the most frequent adverse fetal outcome, followed by IUFD and the need for ICU admission (0.58% each) (Table 2 & Figure 2).

**Table 2:** Incidence of adverse maternal and fetal outcomes after emergency and elective CS at King Abdulaziz Medical City, Riyadh, Saudi Arabia

**Obstetric outcomes**	**Emergency (n = 2886)**		**Elective (n = 1419)**		**Total (n = 4305)**		**Significant difference**
	**No.**	**%**	**No.**	**%**	**No.**	**%**	
*Adverse fetal outcome*^*@*^	159	5.51	59	4.16	218	5.06	*χ*2 = 3.61, df1, p = 0.029**
IUFD	22	2.9	3	0.6	25	0.58	
IUGR	96	3.33	44	3.10	140	3.25	
Neonatal death	5	0.17	3	0.21	8	0.19	
ICU	23	0.80	2	0.14	25	0.58	
Cong. anomaly	14	0.49	10	0.70	24	0.56	
*Adverse maternal outcome*^*@*^	175	6.06	44	3.10	219	5.09	*χ*2 = 17.30, df1, p < 0.001**
ICU	23	0.80	4	0.28	27	0.63	
Ruptured uterus	6	0.21	1	0.07	7	0.16	
Hysterectomy	11	0.38	2	0.14	13	0.30	
Blood transfusion	123	4.26	34	2.40	160	3.72	
Retained placenta	4	0.14	0	0.00	4	0.09	
HELLP syndrome	21	0.73	1	0.07	22	0.51	
Pulmonary embolism	2	0.07	2	0.14	4	0.09	
Renal failure	1	0.03	1	0.07	2	0.05	
Heart failure	2	0.07	0	0.00	2	0.05	
Bowel or bladder injury	10	0.35	2	0.14	12	0.28	
Pelvic abscess	2	0.07	0	0.00	2	0.05	
Maternal death	1	0.03	0	0.00	1	0.02	

@: categories are not mutually exclusive.

*χ*2: Chi-square test, **: Statistically significant difference.

### Significant predictors of adverse maternal/fetal outcomes

Adverse maternal outcomes were significantly predicted in CS by high gravidity, preeclampsia as well as prematurity. Women giving birth to their fourth child were three times (OR = 2.84, 95%CI:1.26-6.39, p = 0.011) more likely to experience adverse maternal outcomes compared to gravida 1 females. This was the same for preeclampsia (OR = 2.84, 95% CI:1.83-4.39, p < 0.001). Females with preterm delivery were significantly more likely to suffer from abnormal maternal outcomes than those females with term newborns OR = 2.39, 95% CI:1.75-3.28, p < 0.001). (Table 3).

**Table 3:** Predictors of adverse maternal outcomes for mothers delivered by CS at King Abdulaziz Medical City, Riyadh, Saudi Arabia

**Characteristics**	**Total (n = 4305)**	**Adverse maternal outcome (n = 219, 5.09%)**		**cOR (95%CI)**	**aOR (95%CI)**
	No.	No.	%	No.	
*Maternal age (years)*					
<20	108	3	2.8	1^@^	1^@^
20-24	666	28	4.2	1.54 (0.46-5.14)	1.26(0.37-4.29)
25-29	1084	47	4.3	1.59(0.49-5.18)	1.27(0.38-4.30)
30+	2447	141	5.76	2.14(0.67-6.82)	1.25(0.37-4.28)
*Gravidity*					
One	849	31	3.7	1^@^	1^@^
Two	651	23	3.5	0.97 (0.56-1.67)	1.47(0.71-3.03)
T*hree*	538	25	4.6	1.29 (0.75-2.20)	2.17(0.95-4.96)
Four or more	2267	140	6.2	1.74 (1.17-2.58)^**^	2.84(1.26-6.39)^**^
*Parity*					
Nulliparity (0 births)	1042	43	4.1	1^@^	1^@^
Multiparity (1-4 births)	2089	100	4.8	1.17(0.81-1.68)	0.60(0.30-1.18)
Grand multiparity(5+ births)	1174	76	6.5	1.61 (1.09-2.36)^**^	0.65(0.31-1.37)
*Gestational age*					
37-42 weeks (term)	3392	134	4.0	1^@^	1^@^
<37 weeks (preterm)	782	80	10.2	2.77 (2.08-3.69)^**^	2.39(1.75-3.28)^**^
>42 weeks (postterm)	131	5	3.8	0.96(0.39-2.39)	1.10(0.44-2.75)
*Twinning*	217	13	6.0	1.20 (0.67-2.14)	0.89(0.53-1.50)
*GDM*	807	46	5.7	1.16 (0.83-1.62)	1.05(0.74-1.49)
*Maternal comorbidity*	620	43	6.9	1.48 (1.05-2.09)^**^	1.14(0.80-1.64)
*Preeclampsia*	209	32	15.3	3.78 (2.52-5.66)^**^	2.84(1.83-4.39)^**^
*Hydramnios*	235	14	6.0	1.19 (0.68-2.09)	1.11(0.63-1.95)

**: Statistically significant difference, @---reference category, cOR---crude odds ratio, aOR---adjusted odds ratio.

Note: every variable was adjusted for all other variables in the table.

Adverse fetal outcomes were predicted in CSs by: twinning, hydramnios, preeclampsia as well as prematurity. Women giving birth to twin were two times (OR = 1.81,95% CI:1.25-2.61, p = 0.002) more likely to experience adverse fetal outcomes compared to singleton women. Pregnant women with hydramnios were sevenfold (OR = 6.70, 95% CI:4.57-9.83, p < 0.001) more likely to have adverse fetal outcomes than those with normal amniotic fluid levels. Women with preeclampsia were significantly three times (OR = 2.74, 95% CI:1.75-4.30, p < 0.001) more likely to have adverse fetal outcomes. Females with preterm delivery were significantly more likely to suffer from abnormal fetal outcomes than those females with term newborns (OR = 4.57, 95% CI: 3.33-6.28, p < 0.001). (Table 4).

**Table 4:** Predictors of adverse fetal outcomes for mothers delivered by CS at King Abdulaziz Medical City, Riyadh, Saudi Arabia

**Characteristics**	**Total (n = 4305)**	**Adverse fetal outcome (n = 218, 5.06%)**		**cOR (95%CI)**	**aOR (95%CI)**
	No.	No.	%		
*Maternal age (years)*					
<20	108	5	4.6	1^@^	1^@^
20-24	666	50	7.5	1.68 (0.65-4.31)	1.39(0.51-3.76)
25-29	1084	62	5.7	1.26 (0.49-3.19)	1.23(0.45-3.34)
30+	2447	101	4.1	0.89 (0.38-2.53)	0.86(0.31-2.40)
*Gravidity*					
One	849	57	6.7	1^@^	1^@^
Two	651	39	6.0	0.89 (0.58-1.35)	1.14(0.59-2.20)
T*hree*	538	22	4.1	0.59(0.36-1.00)	0.94(0.42-2.08)
Four or more	2267	100	4.4	0.64 (0.46-0.90)^**^	1.35(0.62-2.90)
*Parity*					
Nulliparity (0 births)	9.5	72	6.9	1^@^	1^@^
Multiparity (1-4 births)	56.0	100	4.8	0.68 (0.50-0.93)^**^	0.71((0.37-1.36)
Grand multiparity(5+ births)	34.5	46	3.9	0.55(0.38-0.80)^**^	0.61(0.28-1.32)
*Gestational age*					
37-42 weeks (term)	3392	98	2.9	1^@^	1^@^
<37 weeks (preterm)	782	118	15.2	5.99 (4.52-7.92)^**^	4.57(3.33-6.28)^**^
>42 weeks (postterm)	131	2	1.5	0.52 (0.13-2.13)	0.51(0.12-2.12)
*Twinning*	217	36	16.6	1.25 (2.89-6.26)^**^	1.81(1.25-2.61)^**^
*GDM*	807	30	3.7	0.68 (0.46-1.01)	0.66(0.43-1.01)
*Maternal comorbidity*	620	34	5.5	1.12 (0.76-1.61)	0.98(0.65-1.47)
*Preeclampsia*	209	35	16.7	4.28(2.89-6.34)^**^	2.74(1.75-4.30)^**^
*Hydramnios*	235	49	21.1	6.16 (4.34-8.75)^**^	6.70(4.4.57-9.83)^**^

**: Statistically significant difference, @---reference category, cOR---crude odds ratio, aOR---adjusted odds ratio.

Note: every variable was adjusted for all other variables in the table.

## Discussion

CS is medically indicated when a significant risk of an adverse outcome for the mother or fetus is present if the operation is not performed at a given time. In contrast, non-medically indicated CS occurs for reasons other than a risk of adverse outcome [26]. At King Fahd Armed Forces Hospital, which is a tertiary hospital in Riyadh, Saudi Arabia, the CSR has always been below 15%; however, in 2007, the CSR exceeded 20%, a finding that raised concern, as there was no change in the population being served or in the type of practice [27]. In the present study, CS deliveries constituted 19.05% of all deliveries between June 1, 2008, and February 2011 at KAMC. International concern over such increases have prompted the World Health Organization to suggest that the CSR should not exceed 15% [28]; additionally, some evidence has indicated that CSRs above 15% are not associated with additional reductions in maternal or neonatal mortality and morbidity [29].

The determinants of the CSR are likely to be extremely complex. Currently, in the developed world, approximately 30% of CSs are repeat CSs after a primary CS, 30% are performed due to dystocia, 11% are performed due to breech presentation, and 10% are performed due to fetal distress [2,3]. The present study showed similar findings; difficult labor and previous CS were the major indications for CS, followed by fetal distress, beech presentation, maternal condition, multiple delivery and maternal request, whereas cord prolapse and fetal conditions were the least common indications. The data demonstrated that difficult labor, fetal distress, breech presentation and APH were the main indications for emergency CS, whereas previous CS, breech presentation, maternal request and maternal conditions were the main indications for elective CS.

It has become clear that lower segment CS is less likely associated with disastrous ruptures than in the case of classical vertical scar, and the concept of ‘trial of labor” became prevalent in subsequent deliveries. In the present study, more than half of the elective CSs (54.3%) were prompted by a previous CS. This finding was similar to the results of other studies in Saudi Arabia [27,30].The reluctance to permit a trial of labor after previous CS is likely due to a variety of reasons. First, maternal preference likely plays a large part, with CS being regarded as a safe and convenient procedure. Second, the clinician is also likely to regard the CS as a routine, safe, and convenient alternative [31]. There is little work currently available examining the reasons for the clinician's or mother's choice in this area, particularly in communities with high parity, such as the one included in this study, in which more than a quarter (27.3%) of the patients were grand multiparous, with five or more births.

There are well-documented adverse health outcomes associated with cesarean birth, both for the woman and her infant [32-40]. In the present study, adverse maternal outcomes were diagnosed in 219 women, resulting in an incidence rate of 5.09 per 100 CS women. It has been clearly shown that an emergency CS is associated with a substantial rate of morbidity and mortality [41], and the present study showed similar findings. Significant maternal morbidity is often used as a proxy for maternal mortality, as maternal mortality rates, from any cause, are low in developed countries [42]. In our study, HELLP syndrome was identified in 5 out of 1000 CS births and had a significantly higher incidence rate among emergency CSs than elective CSs. This figure was comparable with the 7.1 per 1000 CS births reported by Zwart and colleagues [38]. However, HELLP syndrome and preeclampsia may be conditions leading to CS. Other adverse outcomes included the need for a blood transfusion and ICU admission. In Bergholt's [32] retrospective review of almost 1000 CSs, an estimated blood loss of greater than 1000 ml was recorded in 9.2% of the cesarean births, with 1% of women requiring a blood transfusion. In our study, blood transfusion ranked first as an adverse maternal outcome, with 3.72% of women requiring a blood transfusion. According to the administrative policy and procedure (APP#1427-04, 2013) of the hospital for transfusion of blood and blood components, Red Blood Cells may be transfused into adult patients if any of the following criteria are met: Acute blood loss due to obstetric hemorrhage characterized by the loss of more than 15% of blood volume with evidence of inadequate oxygen delivery; chronic symptomatic anemia of hemoglobin less than 80 g/L in patients with operative procedures associated with major blood loss; or as a preoperative preparation for Sickle Cell Disease patients. Such APP may need to be revised to specify the absolute and the easy to practise indications for blood transfusion, in order to avoid the possible overuse of this practice by health care providers.

In a large study of pregnancy outcomes in Latin American countries conducted by the WHO, CS was associated with an increased risk of maternal death, although the absolute risk of death was low (0.04% for elective CS and 0.06% for emergency CS) [35]. In our study, only one death was reported after emergency CS, resulting in a rate of 0.03%. Gravidity, preeclampsia and preterm delivery were identified as significant risk factors for adverse maternal outcomes. Gravida 4 females were threefold more likely to experience adverse maternal outcomes, when compared with gravida 1 females. Pregnant women with preeclampsia were also threefold more likely to suffer adverse maternal outcomes. With preterm delivery, females were more than two times more likely to suffer from adverse maternal outcomes than with term delivery.

CS deliveries are associated with high risks of respiratory complications, neurological impairment of the newborn, and long-term postpartum morbidity for the mother [43]. Evidence suggests that term infants born by elective CS under regional anesthesia have no greater need for resuscitation than those born vaginally [44-46]. In our study, only two infants were admitted to the ICU (0.14%) following elective CS, whereas 0.80% of infants born after emergency CS were admitted to the ICU. However, these ICU admissions are more commonly amongst preterm infants.

The association of CS with perinatal mortality remains unclear. A reduction in the risk of perinatal deaths has been cited as a potential benefit of cesarean birth [47]. In the present study, intrauterine fetal deaths (IUFD) occurred at a rate of 5.8 per 1000 CS deliveries, and this rate was significantly higher after emergency CS than elective CS. Hopefully such deaths did not occur as a consequence of the procedure, but they might be the consequence of some confounders of emergency CS; such as fetal disress which was the main indication for 21.9% of all emergency CSs. More than one-third of women (37.2%) experienced one or more abortions and/or stillbirths. Moreover, a total of 8 neonatal deaths (1.9 per 1000 CS deliveries) were observed. Adverse fetal outcomes after CS were predicted by preterm delivery, twinning, preeclampsia, and hydramnios. Pregnant women with multiple pregnancy, preeclampsia or hydramnios were two, three and sevenfold respectively, more likely to have infants with adverse fetal outcomes than those with no pregnancy risk. Females with preterm delivery newborns were 4.6 times more likely to have abnormal fetal outcomes than those females with term newborns.

This was a large study conducted on a representative population with a high participation rate, which identified the primary indications for CS among Saudi women at a tertiary care facility. However, due to the retrospective nature of this study, the sensitivity and specificity of the co-morbid diagnoses were variable, and the severity and duration of these diseases were not properly factored into this analysis. Thus, there may be residual related to co-morbidity confounders in this study. No data and therefore no analysis was available of women who have undergone "many" previous Sections, especially in a community of high parity. Moreover, the study was only conducted in one setting and does not represent other health care settings in Saudi Arabia. Thus, these findings cannot be generalized.

## Conclusions

Difficult labor and previous CS were the main indications for CS in Saudi Arabia, a country with high parity and high gravidity. Blood transfusion was the most frequent adverse maternal outcome, while IUGR was the most frequent adverse fetal outcome. Gravidity was a significant predictor of adverse maternal outcomes, and Preterm delivery was a significant predictor of both adverse maternal and fetal outcomes.

Changing the cultural opposition to CS limitations, and encouraging women to consider embarking on fewer pregnancies could act as a safeguard against mandatory CSs for subsequent births in multigravida and grand-multigravidi Saudi females, with further adverse maternal and/or fetal outcomes. APP of transfusion of blood and blood components may need to be revisited. Future prospective study that addresses women with repeat CSs and their association with adverse maternal and fetal outcomes is recommended.

### Details of ethics approval

The study protocol was approved by the Institutional Review Board of the Saudi National Guard Affairs, Riyadh, Saudi Arabia (Ref. #RR07/34).

## Competing interests

The authors report no financial affiliations or other conflicts of interest related to the subject of this study.

## Authors’ contributions

MAR, FAS, and MAA participated in the conception of the study and wrote the study protocol. MAA analyzed the data and drafted the main text of the article, whereas MAR and FAS critically revised the analyses and text for content. MAR planned and supervised the entire study and data collection. The final manuscript has been approved by all authors.

## Pre-publication history

The pre-publication history for this paper can be accessed here:

http://www.biomedcentral.com/1471-2393/14/92/prepub
